# Improving adherence with eye medication: a patient-centred approach to prescribing

**Published:** 2023-05-22

**Authors:** Boateng Wiafe, Nisha Jha, Elmien Wolvaardt, Mohammed Abdull

**Affiliations:** Technical Advisor: Operation Eyesight Universal, Accra, Ghana.; Professor: Department of Clinical Pharmacology and Therapeutics, KIST Medical College and Teaching Hospital, Lalitpur, Kathmandu, Nepal.; Editor-in-Chief: *Community Eye Health Journal*, ICEH, LSHTM, London, UK.; Chief Consultant/Associate Professor: Ophthalmology Department, Abubakar Tafawa Balewa University, Bauchi, Nigeria.

**Managing infectious eye conditions such as ulcers, or chronic conditions such as glaucoma, requires that patients regularly instil eye medication, often for long periods of time. How can prescribers support patients to do so?**

**Figure:**
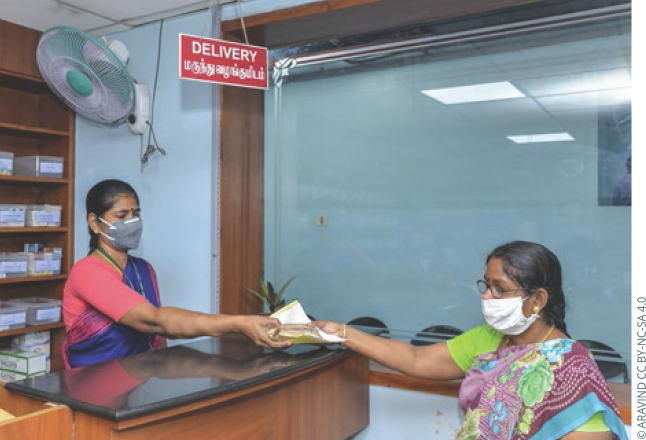
Giving patients the right medicines starts during the prescribing process. india

For eye medications to work, they must be used as prescribed and for as long as needed. In chronic diseases, such as glaucoma, this is for life.

There are many challenges when it comes to medication adherence. These include the availability and affordability of the medication, instilling the medication correctly, remembering when to do it, and doing it always, as is the case with long-term or chronic eye conditions.

Clinicians must therefore carefully consider patients’ ability to adhere to a medication regimen when deciding how to manage their condition. For example:

- Should we admit the patient with a serious corneal infection, or send them home with eye drops?
- If a patient has glaucoma, should we prescribe eye drops to lower intraocular pressure (IOP), or offer surgery/laser?

## What is adherence?

We prefer to use of the term ‘adherence’ instead of ‘compliance’ when talking about how well a patient is able to follow the medication regimen that was agreed between them and the prescriber/clinician.

The term ‘compliance’ is associated with the idea that a patient must follow a set of instructions imposed on them. When health care workers adopt this perspective, patients may be viewed as a passive recipient of health care, and health workers may blame or shame them if they don't use their medication as prescribed.

The term ‘adherence’, however, recognises that a prescription is the result of a joint decision-making process between the patient and the clinician. This recognises the patient as an active participant in their own health care (or a co-producer of health) and acknowledges their right to choose as well as their individual circumstances and challenges. When something goes wrong, and a patient does not adhere to the prescribed medication regimen, health workers who adopt this perspective can work with patients to explore causes and solutions.

“The term ‘adherence’ recognises that a prescription is the result of a joint decision-making process between the patient and the clinician.”

## Prescribing to support adherence

Consider the following aspects when deciding on the best medication regimen for your patients.

- The local **availability** of the medication, and patients’ **ability to pay**. If it is not available locally or is too expensive, consider an alternative medication or other treatment options such as surgery, if appropriate. If it is not covered by health or medical insurance, or not on the list of medicines approved (or provided) by your country's health service, consider advocating for change (see article on page 11). Generic medications are an important alternative where available as they are often cheaper and as effective as the expensive branded products.
- The **complexity** of the patient's overall medication regimen. Ask what other medication a patient is taking, particularly long-term medication, and consider any adverse drug interactions that could lead to the patient discontinuing their eye medication. You can also help patients to remember to use their eye medication, e.g., by suggesting that they instil eye drops at the same time of day as they would normally take their long term medication or time the instillation of medication with routine activities they carry out daily, such as prayer times for muslims.
- **Support networks.** Does the patient have family members or others who can remind them to take their medication, or motivate them to continue when they want to stop? It is always important to counsel a trusted family member, partner or friend on the medications the patient is being given so they can help them maintain adherence by reminding them and helping them to instil the medication.
- Patients’ **physical health**. This can include their ability to administer eye medication, for example, if they suffer from arthritis. Can someone else help them? Find out if there are installation aids available locally, which make it easier for patients with conditions such as arthritis to instil their own drops.
- Patients’ **cognitive health**. Patients with cognitive impairments, dementia, or attention deficit disorder may struggle to remember when to use their eye drops and may require support from others or electronic reminders (e.g., by programming mobile phones to remind them of medication times).

### A note on patient comfort

Stinging sensation or blurring of vision is a common side effect of many eye drops. As this is transient, it is usually better to educate the patient about this possibility and teach them to cope if it is not severe, as changing medication may not be possible, or will be prohibitively expensive. One exception is chlorhexidine, which can be locally manufactured using a buffer solution which reduces stinging sensation (see page 21).

**Q&A: Prescribing glaucoma medication**

**Figure:**
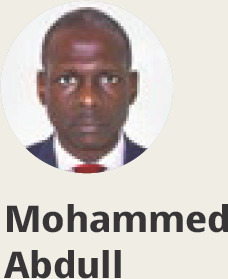


**My preferred choice for most of my glaucoma patients is to prescribe a combination drug treatment, used just once or twice a day.**

**Q. Why a combination drug?**

**A.** It is better to prescribe as few different types of drops as possible, because the chance of poor adherence increases with the number of medications used. Therefore, if multiple medications are needed, it makes sense to use drug combinations: one eye drop bottle that has two or more active ingredients that work together safely and effectively.

**Q. Why less often?**

**A.** It is always preferable to give patients medications that require as few instillations per day as possible. This is because the chance of poor adherence increases with increasing instillation frequency of the drug. So, an eye drop that is used just once a day is usually better adhered to than one that must be used four times a day. In this respect, it makes sense to use slow-release preparations, if available.

